# Epigenetic modelling of former, current and never smokers

**DOI:** 10.1186/s13148-021-01191-6

**Published:** 2021-11-17

**Authors:** Ryan J. Langdon, Paul Yousefi, Caroline L. Relton, Matthew J. Suderman

**Affiliations:** 1 MRC Integrative Epidemiology Unit, University of Bristol, Bristol, UK; 2 Population Health Sciences, Bristol Medical School, University of Bristol, Bristol, UK

**Keywords:** Epigenetic, Smoking, Classification, Methylation, Epidemiology

## Abstract

**Background:**

DNA methylation (DNAm) performs excellently in the discrimination of current and former smokers from never smokers, where AUCs > 0.9 are regularly reported using a single CpG site (cg05575921; *AHRR*). However, there is a paucity of DNAm models which attempt to distinguish current, former and never smokers as individual classes. Derivation of a robust DNAm model that accurately distinguishes between current, former and never smokers would be particularly valuable to epidemiological research (as a more accurate smoking definition vs. self-report) and could potentially translate to clinical settings. Therefore, we appraise 4 DNAm models of ternary smoking status (that is, current, former and never smokers): methylation at cg05575921 (AHRR model), weighted scores from 13 CpGs created by Maas et al. (Maas model), weighted scores from a LASSO model of candidate smoking CpGs from the literature (candidate CpG LASSO model), and weighted scores from a LASSO model supplied with genome-wide 450K data (agnostic LASSO model). Discrimination is assessed by AUC, whilst classification accuracy is assessed by accuracy and kappa, derived from confusion matrices.

**Results:**

We find that DNAm can classify ternary smoking status with reasonable accuracy, including when applied to external data. Ternary classification using only DNAm far exceeds the classification accuracy of simply assigning all classes as the most prevalent class (63.7% vs. 36.4%). Further, we develop a DNAm classifier which performs well in discriminating current from former smokers (agnostic LASSO model AUC in external validation data: 0.744). Finally, across our DNAm models, we show evidence of enrichment for biological pathways and human phenotype ontologies relevant to smoking, such as haemostasis, molybdenum cofactor synthesis, body fatness and social behaviours, providing evidence of the generalisability of our classifiers.

**Conclusions:**

Our findings suggest that DNAm can classify ternary smoking status with close to 65% accuracy. Both the ternary smoking status classifiers and current versus former smoking status classifiers address the present lack of former smoker classification in epigenetic literature; essential if DNAm classifiers are to adequately relate to real-world populations. To improve performance further, additional focus on improving discrimination of current from former smokers is necessary.

**Supplementary Information:**

The online version contains supplementary material available at 10.1186/s13148-021-01191-6.

## Background

Modelling complex phenotypes using DNA methylation (DNAm) is becoming increasingly common in the field of epigenetic epidemiology. This process often includes the use of weighted DNAm “scores” to differentiate between classes of categorical exposures, estimate continuous exposures and predict disease outcomes. A notable advantage of modelling phenotypes using DNAm is that, when validated and applied to external samples, DNAm models can overcome certain limitations of self-reported data collection. Specifically, DNAm models can reduce recall bias as they do not rely on an individual’s recollection of historic information to determine a phenotype. Additionally, because DNAm itself is a continuous measure (0–100%), a categorical phenotype proxied by this biomarker can be represented on the continuous scale [1]. Accordingly, using DNAm proxies for a categorical phenotype can aid epidemiological and clinical research by both classifying categories of the phenotype (using pre-defined thresholds to separate classes), whilst also providing a granular index of phenotype *within* these categories. For example, DNAm models of smoking can be used to determine heavy smokers from never smokers, but can also be used as an index for the *degree* of smoking heaviness in the heavy smokers [2].

Multiple validated DNAm models of smoking have been established which consistently perform well at discriminating current and former smokers from never smokers [3–9]. Distinguishing between current and never smokers can be done almost perfectly using the DNAm status at just 1 cytosine-phosphate-guanine (CpG) site in the aryl hydrocarbon repressor receptor (*AHRR*) gene (cg05575921) [8], which is included in almost all published DNAm models of smoking [10]. For “ever” (that is, current and former smokers combined) versus never smoking, Maas et al. have recently published a weighted combination of 13 CpG sites in peripheral blood which can distinguish between these two smoking classes with an area under the receiver–operator (ROC) curve (AUC) of over 0.90 in external validation data [4].

However, despite the success of classifying current and ever versus never smokers, distinguishing between current and former smokers appears to be much more challenging. Smoking-related DNAm tends to group individuals into three clusters corresponding to current, former and never smokers with the cluster for former smokers appearing somewhere between, and overlapping with, the current and never smoker clusters [11]. The overlap with current smokers is due to individuals having recently quit smoking and the overlap with never smokers due to individuals having not smoked for several years. These overlaps make them difficult to reliably classify, and, in the interest of simplicity, most studies have either excluded former smokers or combined them with current smokers in a group called ‘ever’ smokers.

Despite these challenges, development of a robust DNAm classifier that can accurately distinguish between current, former and never smokers would be valuable for epidemiological research and potentially in clinical settings. We therefore develop several ternary classifiers of current, former and never smokers using Infinium HumanMethylation450 BeadChip (450K) DNAm profiles from the peripheral blood of 1063 European individuals and systematically compare their performances with published models in an independent set of 717 individuals.

## Results

We investigated the discrimination and classification accuracy for ever versus never smoking and current versus former smoking using four respective DNAm models: methylation at cg05575921 (AHRR model), weighted scores from 13 CpGs created by Maas et al. (Maas model), weighted scores from a LASSO model of candidate smoking CpGs from the literature (candidate CpG LASSO model), and weighted scores from a LASSO model supplied with genome-wide 450K data (agnostic LASSO model). Numbers of supplied CpGs versus nonzero CpGs (i.e. retained by the LASSO regression, where appropriate) for each model can be seen in Table 1. Discriminative performance in development and external validation data can be seen in Table 2. Statistics for model classification accuracy can be seen in Table 3 (binary classifiers) and Table 4 (ternary classifiers). Model coefficients, ontological network graphs and enrichment analysis results of constituent CpGs can be seen in Additional file 1: Table S1, Additional file 2: Figures S1–S4, Additional file 2: Tables S2a–s2h, respectively.

**Table 1:** Initial and final numbers of CpGs for each DNAm model of smoking

Classes	Model name	Novel/literature	Number of supplied features to LASSO (CpGs)	Final number of features (CpGs)
“Ever” versus never	AHRR	Literature	NA	1
	Maas	Literature	NA	13
	Agnostic LASSO	Novel	450K	29
	Candidate CpG LASSO	Novel	14	9
Current versus former	AHRR	Literature	NA	1
	Maas	Literature	NA	13
	Agnostic LASSO	Novel	450K	20
	Candidate CpG LASSO	Novel	40	4

“Literature”-based models contain pre-specified CpG sites and betas and were therefore not supplied to LASSO models in this paper. “Novel” models denote models where we supplied sets of CpGs for feature selection via cross-validated LASSO. The “final number of features” are those used to create the various DNAm scores of smoking seen in this paper

**Table 2:**
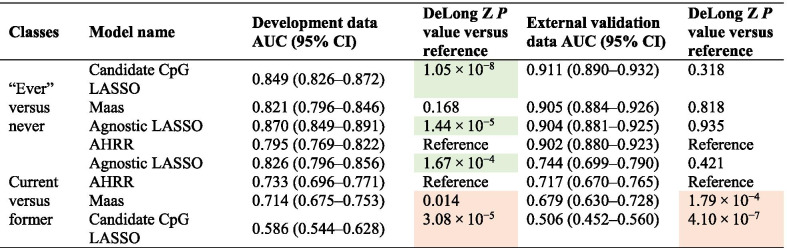
Performance of DNA methylation scores for discrimination between binary smoking statuses

Comparison of the discrimination of DNAm scores for binary smoking status problems, with AHRR model (cg05575921 methylation) as a reference. AUCs were compared to the reference using a DeLong's *Z*-test. Green cells indicate a statistical difference where a classifier improved upon the reference. Orange cells indicate where a classifier performed statistically worse than the reference

**Table 3:** Performances of binary classifiers of smoking status

Data	Accuracy statistics	AHRR model (reference)	Candidate CpG LASSO model	Maas model	Agnostic LASSO model
*Ever/never smokers*					
Training data	Accuracy (95% CI)	0.721 (0.693–0.747)	0.771 (0.744–0.795)	0.752 (0.725–0.777)	0.792 (0.766–0.816)
	NIR (P: Acc > NIR)	0.658 (6.3 × 10^−6^)	0.658 (7.3 × 10^−16^)	0.658 (2.1 × 10^−11^)	0.658 (< 2.2 × 10^−16^)
	Kappa	0.444	0.527	0.480	0.577
	Sensitivity	0.661	0.743	0.745	0.744
	Specificity	0.835	0.824	0.764	0.885
	PPV	0.885	0.890	0.858	0.925
	NPV	0.562	0.625	0.610	0.658
External validation data	Accuracy (95% CI)	0.815 (0.784–0.842)	0.837 (0.808–0.863)	0.822 (0.791–0.849)	0.822 (0.791–0.849)
	NIR (P: Acc > NIR)	0.637 (< 2.2 × 10^−16^)	0.637 (< 2.2 × 10^−16^)	0.637 (< 2.2 × 10^−16^)	0.637 (< 2.2 × 10^−16^)
	Kappa	0.624	0.661	0.627	0.633
	Sensitivity	0.766	0.818	0.814	0.792
	Specificity	0.900	0.869	0.835	0.873
	PPV	0.931	0.917	0.896	0.917
	NPV	0.686	0.731	0.719	0.705
*Current/former smokers*					
Training data	Accuracy (95% CI)	0.707 (0.671–0.740)	0.512 (0.474–0.550)	0.700 (0.664–0.733)	0.757 (0.723–0.788)
	NIR (P: Acc > NIR)	0.522 (< 2.2 × 10^−16^)	0.522 (0.715)	0.522 (< 2.2 × 10^−16^)	0.522 (< 2.2 × 10^−16^)
	Kappa	0.416	0.025	0.403	0.516
	Sensitivity	0.658	0.504	0.625	0.701
	Specificity	0.761	0.521	0.781	0.817
	PPV	0.750	0.535	0.758	0.808
	NPV	0.670	0.490	0.656	0.715
External validation data	Accuracy (95% CI)	0.646 (0.600–0.689)	0.541 (0.494–0.587)	0.619 (0.573–0.664)	0.674 (0.629–0.717)
	NIR (P: Acc > NIR)	0.576 (1.3 × 10^−3^)	0.576 (0.940)	0.576 (0.03)	0.576 (9.9 × 10^−6^)
	Kappa	0.318	0.093	0.251	0.373
	Sensitivity	0.825	0.603	0.706	0.861
	Specificity	0.513	0.494	0.555	0.536
	PPV	0.556	0.468	0.539	0.578
	NPV	0.799	0.628	0.719	0.839

**Table 4:** Performance of ternary classifiers of smoking status (current, former and never)

Data	Accuracy statistics	AHRR model (reference)	Candidate CpG LASSO model	Maas model	Agnostic LASSO model
Training data	Accuracy (95% CI)	0.606 (0.576–0.635)	0.538 (0.508–0.568)	0.619 (0.589–0.648)	0.695 (0.667–0.723)
	NIR (P: Acc > NIR)	0.364 (< 2.2 × 10^−16^)	0.364 (< 2.2 × 10^−16^)	0.364 (< 2.2 × 10^−16^)	0.364 (< 2.2 × 10^−16^)
	Kappa	0.405	0.306	0.427	0.541
	*Never smokers*				
	Sensitivity	0.835	0.824	0.764	0.885
	Specificity	0.661	0.743	0.745	0.744
	PPV	0.562	0.625	0.610	0.643
	NPV	0.885	0.890	0.858	0.925
	*Former smokers*				
	Sensitivity	0.299	0.380	0.455	0.518
	Specificity	0.872	0.763	0.797	0.892
	PPV	0.518	0.423	0.507	0.687
	NPV	0.731	0.729	0.762	0.802
	*Current smokers*				
	Sensitivity	0.658	0.397	0.625	0.669
	Specificity	0.875	0.802	0.887	0.905
	PPV	0.730	0.512	0.743	0.787
	NPV	0.830	0.720	0.819	0.839
External validation data	Accuracy (95% CI)	0.612 (0.576–0.648)	0.594 (0.557–0.609)	0.603 (0.566–0.639)	0.637 (0.601–0.673)
	NIR (P: Acc > NIR)	0.367 (< 2.2 × 10^−16^)	0.367 (< 2.2 × 10^−16^)	0.367 (< 2.2 × 10^−16^)	0.367 (< 2.2 × 10^−16^)
	Kappa	0.405	0.390	0.406	0.462
	*Never smokers*				
	Sensitivity	0.900	0.869	0.835	0.873
	Specificity	0.766	0.818	0.814	0.792
	PPV	0.686	0.731	0.719	0.705
	NPV	0.931	0.917	0.896	0.917
	*Former smokers*				
	Sensitivity	0.171	0.368	0.297	0.270
	Specificity	0.914	0.811	0.824	0.916
	PPV	0.536	0.530	0.494	0.651
	NPV	0.656	0.689	0.669	0.684
	*Current smokers*				
	Sensitivity	0.825	0.531	0.706	0.820
	Specificity	0.748	0.767	0.771	0.757
	PPV	0.548	0.458	0.533	0.556
	NPV	0.920	0.815	0.876	0.919

N.B. Ternary classifiers are the result of two binary classifiers being applied to DNAm data in sequence: ever versus never smoker classification, then current versus former classification of the ever smokers

### Classifier performance

New classifiers developed using LASSO each included a DNAm score created from between 4 and 29 CpGs. For each, this number was comfortably below the 61 and 42 parameters calculated as our theoretical maximum for the ever/never and current/former DNAm classification scores, respectively.

The best-performing score for discriminating between ever and never smokers was the candidate CpG LASSO score with an AUC of 0.911 in external validation data (95% CI 0.89–0.932). However, this performance was indistinguishable from our AHRR reference (AUC 0.902, 95% CI 0.88–0.923). For current/former smokers, the best-performing score was the agnostic LASSO classification score, with an AUC of 0.744 (95% CI 0.699–0.790). However, when compared to the AHRR reference (AUC: 0.717; 95% CI 0.670–0.765), there only a slight improvement in discrimination is seen for this classifier. In fact, no single binary classifier notably improved upon our AHRR model reference, with the Maas score and candidate CpG LASSO score both performing statistically worse at discriminating current versus former smokers.

After converting scores to classifiers by generating optimised thresholds, the candidate CpG LASSO classifier demonstrated the highest accuracy in external validation data for ever versus never smokers (83.7%, 95% CI 80.8–86.3%). However, this accuracy was not distinguishable from that of our AHRR reference, which was 2.2% lower (81.5% 95% CI 78.4–84.2%). The same trend was seen when converting DNAm classification scores to classifiers for current versus former smokers; the agnostic LASSO classifier showed the highest accuracy in external validation data (67.4%, 95% CI 62.9–71.7%), but did not noticeably outperform our AHRR reference (64.6%, 95% CI 60.0–68.9%).

Having derived and evaluated classifiers for the binary classification problems, we then derived ternary classifiers that applied two binary classifiers consecutively. Here, the agnostic LASSO had greatest accuracy in both our development and external validation data, at 69.5% (95% CI 66.7–72.3%) and 63.7% (95% CI 60.1–67.3%), respectively. These were an improvement of 8.9% (ever vs. never) and 2.5% (current vs. former), respectively, over using the AHRR classifiers.

### Gene set enrichment analysis results of CpGs in smoking classifiers

We performed enrichment analysis of the genes mapped to CpGs in our DNAm models. For genes associated with our agnostic LASSO ever versus never smoker score (*N* = 21), BioPlanet 2019 (biological) pathways showed enrichment for haemostasis, platelet homeostasis, G alpha (s) signalling events, chromatin remodelling by nuclear receptors to facilitate initiation of transcription in carcinoma cells, and the rapid glucocorticoid receptor pathway. Human Phenotype Ontologies (HPOs) were largely enriched for stress and obesity, including agitation, striae distensae, hypercortisolism, restlessness, and truncal obesity. For genes mapped to CpGs associated with our agnostic LASSO current versus former score (*N* = 14), BioPlanet 2019 pathways showed enrichment for molybdenum cofactor biosynthesis, facilitative sodium-independent glucose transporter, class C G-protein-coupled receptors (GCPRs), metabolism of vitamins and cofactors, and the activator protein-1 (AP-1) transcription factor network. HPOs for this score were largely enriched for psychosocial abnormalities and neurodegeneration, including axonal loss, opisthotonus, progressive neurologic deterioration, delayed gross motor development, abnormal social behaviour, and impaired social interactions.

There were five genes (*AHRR, ALPPL2, F2RL3, GNG12* and *PRSS23*) which mapped to our candidate CpG LASSO ever versus never smoker score, comprised of nine CpGs. These genes did not appear to be enriched for any HPOs. However, BioPlanet 2019 pathways showed enrichment for thrombin signalling, platelet activation, folate biosynthesis, G-protein activation and presynaptic function of kainite receptors. No HPO or biological process were found to be associated with the three genes (*TMEM51*, *LOC100128288* and *LINGO3*) which mapped to the four CpG sites from candidate CpG LASSO current versus former score.

Both our AHRR model and Maas model did not use different CpGs for ever versus never smoker and current versus former smoker classification. The AHRR model corresponds to a single gene—*AHRR.* The protein encoded by this gene participates in the aryl hydrocarbon receptor (AhR) signalling cascade, which mediates dioxin toxicity, and is involved in regulation of cell growth and differentiation. It functions as a feedback modulator by repressing AhR-dependent gene expression. The Maas model contained 13 CpGs which mapped to 5 unique genes. These genes are enriched for BioPlanet 2019 biological processes including inhibition of platelet activation by aspirin, thrombin signalling through protease-activated receptors, Myc repressed pathway, tumour necrosis factor-alpha effects on cytokine activity, cell motility and apoptosis, and peptide GPCRs. HPOs were enriched for B lymphocytopenia, abnormality of B cell number, acute myeloid leukaemia and neutropenia.

## Discussion

In this study, we appraised the discrimination and classification accuracy of four DNAm models, consisting of between 4 and 29 CpGs with optimised classification thresholds. We provide evidence that DNAm models are capable of ternary classification of smoking status (current, former and never smoking) with classification accuracy statistics greatly improving upon the NIR in both development and external validation data. Notably, we develop a DNAm classification score that can discriminate well between current and former smokers with methylation status using 21 CpGs (our agnostic LASSO current vs. former smoker classifier), producing an AUC in training data of 0.826 and an AUC in external validation data of 0.744.

All but one of the models appraised in this paper shows evidence of an ability to determine smoking status better than the NIR—assigning every smoking class as the most prevalent observed class. Other than the candidate CpG LASSO binary classifier for current versus former smoking, all binary and ternary classifiers significantly improved on the NIR in development and external validation data. Sixteen of 24 *P* values for the comparison between classification accuracy and NIR were below 2.2 × 10^−16^, indicating a vast accuracy improvement. AUCs for binary smoking DNAm scores show a similar improvement. All DNAm scores but the candidate CpG LASSO current versus former DNAm score showed an AUC (and lower 95% CI bound) above 0.5. For reference, an AUC of 0.5 is considered to be the result of a predictor which makes random class assignments, akin to “chance assignment”.

In order to assess where our classifier may add value in the wider context of biomolecular smoking assessment, we compared the performance of DNAm at CpG sites from our ternary classifier to serum cotinine measurements in the Accessible Resource for Epigenomics Studies (ARIES) [12]—a subset of the Avon Longitudinal Study of Parents and Children (ALSPAC) [13, 14]. DNAm and serum cotinine have both shown excellent performance (AUC > 0.9) when discriminating between current and never smokers [8, 15]. However, the half-life of cotinine is ~ 14 h [16], whereas some smoking-related DNAm signals can persist for over 30 years. Accordingly, for ternary smoking classification (i.e. smokers in a “real-world” population), cotinine may be unable to distinguish between former and never smokers accurately. Indeed, Zhang et al. report that DNAm and cotinine can distinguish current from never smokers with similar accuracy, but that only DNAm can distinguish between former and never smokers with high accuracy. Findings from the comparison of these two biomarkers in ARIES corroborate those of Zhang et al.; AUCs for former versus never smoking were < 0.5 for cotinine, but almost 0.7 for DNAm (Additional file 3: Fig. S5). It should be noted that the ARIES population were pregnant women of mean age 29.2 years old; thus, their exposure to smoking was relatively low compared to older, mixed sex populations. In the older individuals whose samples are used in the current paper (mean age 47; likely, therefore, to have more exposure to smoking), we saw even larger DNAm differences between former and never smokers (AUC > 0.8; Additional file 3: Fig. S5). Such findings underscore a key advantage of DNAm over cotinine as a biomarker, particularly for the classification of former smokers in a given population—certain smoking-related signals remain methylated for a long time after smoking cessation, allowing former smokers to be discriminated from never smokers, but other DNAm signals can revert to “never” smoker levels quickly, allowing former smokers to also be distinguished from current smokers.

One of the limitations of our study is that there may be a systematic difference in smoking characteristics between the development and external validation data, despite an even class distribution in both. When examining the discriminative ability (by AUC) of our DNAm scores, in ever/never smokers AUCs systematically improve from development to external validation data. However, when assessing current/former smoker AUCs, the opposite change occurs and discriminative ability between development and external validation data appears to systematically attenuate. This may suggest that in the development data, there are former smokers who have quit for longer or historically smoked less, leading to less pronounced differences in the methylation profiles of “ever” and never smokers as compared to the external validation data. Contrarywise, with the external validation data, there may be former smokers who have quit more recently or smoked more heavily prior to quitting, thus making current and former smoker methylation profiles look more similar than if they had smoked less or quit earlier, compared to the development data. Whilst a limitation insofar as it may highlight a lack of classifier robustness to differential smoking behaviours, this characteristic of our classifiers may also allow for estimation of the broad proportions of current, former and never smokers between two populations, particularly in absence of phenotypic data. However, this hypothesis necessitates further exploration before it is validated, in a dataset with time since cessation and smoking heaviness phenotype data.

Two CpGs overlapped between our candidate CpG LASSO model, Maas model, and agnostic LASSO DNAm scores for ever versus never smoking: cg06126421 and cg05951221, annotating to 6p21.33 and ALPPL2, respectively. ALPPL2 is responsible for C-terminal protein lipidation, whilst the 6p21.33 locus contains genes associated with sustained smoking and tumorigenesis in the literature. The candidate CpG LASSO DNAm score for ever versus never smoking contained only these two CpG sites and performed marginally better than all other models in both development and external validation data, indicating both the Maas et al. and agnostic LASSO classifiers (though performing well) may generate slight classification error due to unnecessary additional parameters.

For current versus former smoker DNAm score CpGs, only the Maas model and agnostic LASSO model shared a common feature; both contained our reference AHRR model CpG—cg05575921. The agnostic LASSO model score contained 20 CpG sites and showed a nominal increase in AUC over that of our reference AHRR model, whereas the Maas model DNAm score showed a slight decrease in AUC versus the AHRR model. These findings suggest that cg05575921 captures a relatively large proportion of variance in the current and former smoking classes. The Maas model is derived from a systematic appraisal of published smoking EWAS. Given most of these studies investigate ever versus never or current versus never smoking, it is plausible that the Maas model is more specific to the resolution of these classes in particular, and that very few of the 13 sites which comprise this classifier capture any meaningful variation between current and former smokers specifically. Conversely, the agnostic LASSO was developed in data restricted to current and former smokers, thus may contain CpGs which explain more variance in these classes and can distinguish them apart more easily. Finally, the candidate CpG LASSO current versus former DNAm score CpGs did not include cg05575921 and performed substantially worse than the other classifiers. The four CpGs retained from the candidate CpG LASSO of current versus former smoking came from a collection of 40 CpGs, pertaining to “fast” (< 5 years) reversion to never smoker methylation levels in former smokers in Guida et al. [17]. The poor performance seen for this score may reflect a lack of cg05575921 methylation to explain a large proportion of phenotypic variance between current and former smokers. Alternatively, it may reflect a similarity of methylation levels at these sites between current and former smokers in our data versus the discovery cohort of Guida et al. or perhaps our hypothesis that “fast” reversion of CpG sites to never smoker levels can aid distinction of current from former smokers is simply incorrect and requires revisiting.

Our enrichment analyses investigated biological processes associated with constituent CpGs of our DNAm models to interrogate their biological relevance as predictors of smoking; particularly relevant for our agnostic LASSO model, which was supplied genome-wide methylation data. For CpGs in current versus former DNAm score from this model, the most-associated biological process across our enrichment analyses was “molybdenum cofactor biosynthesis”. Molybdenum is present in tobacco smoke [18, 19] and has been shown to be significantly elevated in smokers compared to non-smokers [20]. Biosynthesis of molybdenum cofactors also correlates with increasing circulating levels of this metal [21, 22]. When we investigated CpGs annotated to the genes in our current versus former smoker gene set, there was a single CpG (cg26505878) which annotated to the *MOCS2* gene (Molybdenum Cofactor Synthesis 2). This CpG is located at the same genomic position (chr5:52,405,886; hg19) as binding sites for four transcription factors associated with the *MOCS2* gene (*STAT1, EGR1, TFAP2C* and *RBL2*), perhaps providing evidence that this CpG helps to regulate transcription of *MOCS2* and, by extension, biosynthesis of molybdenum cofactors in response to elevated molybdenum from cigarette smoke.

The most-associated biological process ontology for CpGs in the ever versus never smoker DNAm score from our agnostic LASSO model was “haemostasis pathway”. CpGs mapping to *PDE11A* (cg02369725), *GNAS* (cg03821543), *GP5* (cg13185177) and *F2RL3* (cg03636183) were jointly associated with this particular enrichment term. Cigarette smoke is a known risk factor for cardiovascular disease and has been shown to alter the balance of antithrombotic, prothrombotic, profibrinolytic and antifibrinolytic factors [23]. The balance of these factors appears to be altered due to cigarette smoke affecting the functions of endothelial cells, platelets, fibrinogen, and coagulation factors [24]. McEvoy et al. found that, in data from 6814 participants from the Multiethnic Study of Atherosclerosis, hazard ratios (HRs) for all-cause cardiovascular disease were 1.4 (95% CI 1.2–1.8) for current smoker versus never smokers, and 1.3 (95% CI 1.1–1.5) for former smokers versus never smokers [25]. Further, in a prospective study of 188,167 healthy individuals from the 45 and Up Study, from 2006 to 2015, current and past smokers showed statistical evidence of elevated risk of five major cardiovascular disease outcomes versus never smokers [26]. Accordingly, CpGs from our agnostic LASSO model appear to show evidence of biological plausibility related to both current versus former and ever versus never smoking, increasing our confidence that this previously unpublished model can translate across well to new data.

Agreement and disagreement between DNAm-classified smoking and self-report smoking may be an epidemiologically relevant measure. The huge breadth of individual habits in relation to smoking will undoubtedly affect an individual’s risk of future disease, even *within* the current, former and never smoking statuses. For example, current smokers who smoke more cigarettes per day than other current smokers, former smokers who quit more recently than other former smokers, and never smokers who are exposed to second-hand smoke or pollution more regularly than other never smokers may all have an increased risk of lung cancer. To this end, DNAm may be used to identify high-risk individuals within smoking classes. If a self-reported former smoker is classified by DNAm as a current smoker, they may have recently quit or smoked more heavily when they did. If a self-reported never smoker is classified by DNAm as a former or current smoker, they may live in an area of high pollution or be exposed to a high amount of passive cigarette smoke. Ultimately, the lack of perfect agreement between DNAm and self-report may prove a useful artefact if the former were used to augment the latter in epidemiological studies, particularly given the high amount of phenotypic variance DNAm explains in smoking.

Finally, given the good performance statistics and evidence for classifier generalisability shown in this paper, a potentially lucrative avenue for future research exists in applying DNAm proxies of smoking to health outcomes. DNAm proxies of smoking may increase the phenotypic variation explained by smoking in a given health outcome (possibly independently of self-report), making DNAm a key consideration for improving the current definition of smoking. This is particularly important for association studies and prediction models, where accuracy of exposure measurement is critical for correct interpretation of results.

## Conclusions

In summary, DNAm models of smoking can be used to determine ternary smoking status (current, former and never smoking) significantly better than chance assignment. Further, DNAm scores can discriminate excellently between ever and never smokers and reasonably well between current and former smokers. The good performance of DNAm models seen in this paper likely reflects the large amount of phenotypic variance DNAm explains in smoking. Accordingly, a future application of the work here may be that DNAm will be able to augment self-report smoking status to improve the prediction of future disease. We have retained in our work that cg05575921 (AHRR) methylation is an excellent biomarker of smoking status, even when assessing three classes. We have been able to improve slightly on the performance of this well-established CpG; our agnostic LASSO model-derived classifier of smoking showed a marked improvement in classification accuracy in development data and slightly better in external validation data. The novel agnostic LASSO model we developed showed enrichment for biologically plausible smoking pathways and feasible phenotype ontologies, such as haemostasis, molybdenum cofactor synthesis, body fatness and social behaviours. Determining multiclass smoking may be improved on what has been achieved here by proxying characteristics of former smoking which allow them to be distinguished from current and never smokers more readily, such as time since cessation, or perhaps a specific biological pathway such as nicotine withdrawal.

## Methods

### Gene expression omnibus methylation datasets

The dataset used to generate and appraise our DNAm-based smoking classifiers was drawn from four published, peer-reviewed epigenome-wide association studies (EWAS) (total *N* = 1780) with available smoking phenotype and Infinium HumanMethylation450 BeadChip data in the GEO DataSets (https://www.ncbi.nlm.nih.gov/gds) database: Liu et al. [27] (GSE42861), Su et al. [28] (GSE85210), Tsaprouni et al. [29] (GSE50660) and Ventham et al. [30] (GSE87648). Briefly, the Liu et al. dataset comprises 689 individuals, mean age 51.9, from the Epidemiological Investigation of Rheumatoid Arthritis (EIRA) cohort [31], with current, former never smoker statuses determined by self-report questionnaire (*N* = 228, 266 and 193, respectively). The Su et al. dataset consists of 253 healthy individuals, mean age 34.5, who were current and never smokers (*N* = 172 and 81, respectively; 0 former smokers) based on self-report questionnaire in a US population. The Tsaprouni et al. dataset contains 464 individuals, mean age 55.4, from the CARDIOGENICS consortium, based on a self-report questionnaire. Finally, the Ventham et al. dataset consists of 383 individuals, mean age 36.7, from the Inflammatory Bowel Disease Biomarkers Programme (IDB-BIOM), based on self-report questionnaire. Summaries of contributing consortium, GEO accession, sample size, age, sex and smoking status distribution for each study are shown in Table 5. Raw betas for each study were downloaded from GEO using the *GEOquery* R package [32] and were subsequently normalised using functional normalisation, via the *meffil* R package [33].

**Table 5:** Summaries of contributing publicly available studies

Publication	Liu et al.	Su et al.	Tsaprouni et al.	Ventham et al.	Overall
Consortium	EIRA	N/A	CARDIOGENICS	IBD-BIOM	–
GEO accession	GSE42861	GSE85210	GSE50660	GSE87648	–
N	689	253	464	383	1789
Mean age (SD)	51.9 (11.8)	34.5 (8.8)	55.4 (6.7)	36.7 (14.2)	47.0 (13.8)
Gender	492 female; 197 male	82 female; 171 male	137 female; 327 male	184 female; 199 male	895 female; 894 male
Never smokers	193	81	179	171	624
Current smokers	228	172	22	99	559
Former smokers	266	0	263	106	597

For model development, Liu et al. (GSE42861) and Ventham et al. (GSE87648) were combined as training data (*N* = 1063; 364 current, 334 former, 365 never smokers), whilst Tsaprouni et al. (GSE50660) and Su et al. (GSE85210) were combined as external validation data (*N* = 717; 260 current, 263 former, 194 never smokers). This combination of studies allowed an approximately equal weighting of current, former and never smoking classes during both development and validation.

### DNA methylation classifiers of smoking status

We developed four individual DNAm classifiers of ternary smoking status (current, former and never): an AHRR classifier, a Maas classifier, an agnostic LASSO classifier and a candidate CpG LASSO classifier, each of which is described below. We used feature selection via least absolute shrinkage selection operator (LASSO) regression to produce linear outputs of the most informative CpG sites and effect sizes for the agnostic LASSO and candidate CpG LASSO classification scores. The AHRR and Maas classifiers were published, robust smoking classifiers which we applied to our DNAm data.

**Fig. 1:**
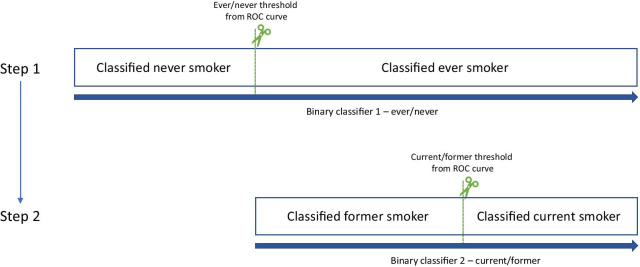
Diagrammatic view of two-stage approach to ternary smoking status classification

All ternary classifiers first distinguished ever from never smokers using a DNAm score with an optimised threshold and then distinguished current from former smokers within the identified ever smokers using a different DNAm score and optimised threshold. That is to say, our ternary smoking classifiers were a combination of two binary smoking classifiers, applied consecutively (Fig. 1). Prior to developing these classifiers, given a fixed sample size for classifier development, we estimated the maximum number of classifier parameters (in this instance, CpG sites) using criteria proposed by Riley et al. [34] These criteria minimise overfitting and thereby improve classifier performance when applied to new individuals.

DNAm classification scores for both binary classifiers needed to satisfy the following criteria proposed for a binary outcome:

1. The DNA scores should each ensure an expected global shrinkage factor (a measure of overfitting from 0 to 1, where higher numbers indicate smaller overfitting) of 0.9 or higher.
2. There should be a small absolute difference (≤ 5%) in the apparent and adjusted Nagelkerke’s R^2^ for each DNAm score. As an estimate for apparent R^2^ in our model, we used 60.9% as reported by McCartney et al. 2018 [6] for their ever versus never smoking model.
3. Each DNAm score should allow for precise estimation (a margin of error ≤ 0.05) of the average proportion of our outcome in the population. In this instance, they should allow for precise estimation of the proportion of ever versus never smokers in our first binary classifier, and the proportion of current versus never smokers in our second binary classifier, in their respective null models (i.e. at the intercept). In our first binary classifier, our outcome proportion of ever smokers was 66%; in our second binary classifier, 52% of individuals were current smokers versus former smokers.

The smallest number of calculated parameters (CpGs) across all three criteria above would satisfy them all. Our development sample sizes were 1063 for ever versus never smoking and 698 for current versus former smoking. Using the *pmsampsize* R package developed by Riley et al. [35], for ever versus never smoking, the theoretical maximum number of CpG sites which satisfied the criteria was 61. For current versus former smoking, the theoretical maximum number of CpG sites which satisfied the criteria was 42.

#### LASSO-derived classification scores

Our “agnostic LASSO” and “candidate CpG LASSO” smoking classification scores were both derived using LASSO regression via the *glmnet* R package [36] in R (version 4.0.3). For the agnostic LASSO classifier, we supplied all available 450K methylation data as independent variables to a cross-validated LASSO (k-folds = 5). This process was repeated independently for ever versus never smokers and current versus former smokers (see Fig. 1 above). Out of a sequence of 100 automatically generated lambda values, the value which produced the minimum mean cross-validated error was selected and used. At this minimum lambda value, our LASSO output for ever versus never smokers returned 29 CpGs, which was fewer than our pre-calculated maximum number of parameters (61 CpGs). The LASSO output for current versus former smokers also contained fewer CpGs than our calculated maximum (20 CpGs versus our 42 CpG maximum).

For our candidate CpG LASSO, we hypothesised that CpG sites which do not revert to never smoker levels (following smoking) could discriminate well between ever and never smokers, whilst CpG sites which revert within a short timeframe could then discriminate well between current and former smokers. Joehanes et al. [9] and Guida et al. [17] are the largest EWAS using 450K data to investigate CpG sites that revert to never smoker levels of DNAm within 5 years of smoking cessation, in addition to CpG sites which do not revert to never smoker levels of DNAm up to 30 years of smoking cessation. Therefore, for ever versus never smoking, we supplied overlapping CpG sites between Joehanes et al. and Guida et al. which do not revert to never smoker levels after 30 years of smoking cessation (*N* = 14) as independent variables to a cross-validated LASSO regression (k-folds = 5). Separately, for current versus former smokers, we supplied overlapping CpGs as independent variables if they reverted to never smoker levels within 5 years of smoking cessation (*N* = 40). Details of all CpGs supplied these regression models can be seen in Additional file 4: Tables S3a and S3b. At the minimum lambda value, both regression outputs contained fewer than our pre-calculated maximum number of CpGs. Our ever versus never LASSO returned nine CpGs (versus a maximum of 61 CpGs) and our current versus former LASSO returned four CpGs (vs. a maximum of 42 CpGs).

DNAm classification scores were constructed from all respective LASSO regression outputs by extracting the CpGs with nonzero coefficients and creating a weighted score in our DNAm data. Weighted scores for each individual in our data were generated by taking the sum of normalised DNAm values at these nonzero CpGs multiplied by the corresponding LASSO beta values.

#### Classification scores from literature

Both normalised AHRR methylation and the Maas classifiers both show published evidence of excellent performance when classifying smoking status. For AHRR, normalised methylation at cg05575921 has been established as a powerful biomarker for classification of smoking status and prediction of smoking-related health outcomes [2, 5, 37, 38]. Our AHRR classifier simply involved using normalised methylation at AHRR to separate ever from never smokers, then current from former smokers. Given the prevalence of literature reporting high performance for this biomarker, we used normalised DNAm at AHRR as our reference classifier (see *Evaluating classifier performance* below).

Elsewhere, Maas et al. recently identified a 13 CpG classifier by employing 14 EWAS for marker discovery, using data from six population-based cohorts (*N* = 3764) from the Biobank-based Integrative Omics Study (BIOS) Consortium for model building [4]. The authors achieved an AUC of 0.901 for “smoking versus non-smoking”, with an AUC in an independent (external) population-based cohort (*N* = 1608) of 0.911. As the largest, most recent systematic attempt at developing a smoking status classifier, we created DNAm classification scores in our data using the CpGs and effect sizes from Maas et al. using the approach outlined in Box 1.

**Box 1:** DNAm classification score generation using Maas et al. stepwise regression data

For each individual in our DNAm data, a weighted score was obtained by multiplying the normalised methylation value at a given CpG by the effect size Maas et al. then summing these values:	
	*b*_1_cpg_1_ + *b*_2_cpg_2_ + … + *b*_13_cpg_13_
where “cpg” is the normalised methylation value in our dataset and “*b*” is the effect size from Maas et al. Additional file 4: Table S3	

#### Generating classification thresholds for smoking status classification

After creating continuous output for ever versus never and current versus former smokers in the form of DNAm classification scores, we sought to distinguish these classes from the scores by creating optimised classification thresholds. For ever versus never smokers, an “ever versus never” binary variable was created in our phenotype data from by combining current and former smokers as “cases” (coded as “1”), whilst leaving never smokers as “controls” (coded as “0”). For current versus former smokers, our phenotype data were restricted to current and former smokers. We created a binary variable where current smokers were “cases” (coded as “1”) and former smokers were “controls” (coded as “0”). Using the *pROC* R package [39] in our development data, we plotted ROC curves of self-report smoking status as the response variable, against each respective DNAm classification score as the predictor variables. From the ROC curves, we extracted the threshold (which can be thought of as a DNAm classification score cut-point) which minimised the Euclidean distance between the ROC curve and the [0, 1] point. It is this threshold which was used to separate “cases” from “controls” in our various DNAm classification scores.

Our ternary classifiers consisted of two DNAm classification scores, alongside two optimised thresholds. Using the ever versus never thresholds, the ever versus never DNAm classification scores were separated into ever versus never smokers. Next, in those classified as ever smokers, we used the current versus former DNAm classification scores to separate these individuals into current and former smokers, using the current versus former thresholds.

### Evaluating classifier performance

We use two definitions for our classifier outputs: “DNAm classification score” and “classifier”. “DNAm classification score” refers to the sum of weighted methylation values for each individual. “Classifier” refers to the separation of the DNAm classification score into respective classes using the optimised thresholds outlined above. For each of our four classifiers, we compared the performance of five objects:

1. The ever versus never smoker DNAm classification score
2. The current versus former smoker DNAm classification score
3. The ever versus never smoker classifier
4. The current versus former smoker classifier
5. The overall ternary classifier of smoking status (current, former and never smoking)

For points 1 and 2 above, we determined the AUC of DNAm scores associated with our binary classifiers against self-report smoking status using the *pROC* R package in our development data. As mentioned previously, due to the high performance and frequency of cg05575921 (*AHRR*) in the literature, we used our AHRR model as a reference, comparing the AUC between all other ever versus never smoker classifiers and all other current versus former smoker classifiers, respectively, to this model, using a DeLong *Z*-test to determine whether they were statistically different.

For points 3–5 above, we used the optimal thresholds to determine smoking classes in our development data, from which we constructed confusion matrices of predicted versus actual smoking status using the *caret* R package [40]. From the confusion matrices, we calculated accuracy, the “no-information rate” (NIR, the largest proportion of the observed classes, used as a comparison against accuracy), unweighted Kappa, sensitivity, specificity, positive predictive value (PPV) and negative predictive value (NPV).

The classifiers generated from our development data were applied to external validation data in order to assess their generalisability and whether there was evidence of overfitting. In external validation data, the same statistics were derived as for the development data above: the AUC for DNAm classification scores, in addition to NIR, unweighted Kappa, sensitivity, specificity, PPV and NPV of classifiers. The thresholds which separated smoking classes in our training data were not recalculated in external validation data; they were applied directly to it.

### Enrichment analysis

CpG were annotated to genes using the Illumina 450K manifest in the *meffil* R package [33]. We then used the Enrichr online platform [41, 42] (https://maayanlab.cloud/Enrichr/#) to compare supplied gene s to its existing database of annotated gene set objects (representing prior biological knowledge) to check for significant overlap. During our enrichment analysis, we used the Human Phenotype Ontology (HPO) [43] and the BioPlanet 2019 integrated biological pathway resource [44] as our reference annotated gene sets.

## Supplementary Information

**Additional file 1**. **Supplementary Table 1** - LASSO model constituent CpGs, betas and gene symbol information.

**Additional file 2**. **Supplementary Tables 2A-2H** - Supplementary Tables displaying results from enrichment analyses of genes mapped to CpGs from the smoking models presented in this paper. **Supplementary Figures 1-4** - Network graphs of Human Phenotype Ontologies associated with CpGs from the smoking models presented in this paper.

**Additional file 3**. **Supplementary Figure 5** - Comparison of the discriminative ability of cotinine and DNA methylation when distinguishing former from never smokers.

**Additional file 4**. **Supplementary Tables 3a and 3b** - Information pertaining to the CpG sites supplied to the ever/never and current/former candidate CpG LASSO models, respectively.

## Data Availability

All results presented from this analysis were conducted using publicly available data. These data can be found on the GEO Datasets Database under the following Dataset IDs: Liu et al.: GSE42861. Su et al.: GSE85210. Tsaprouni et al.: GSE50660. Ventham et al.: GSE87648.
