# Supplementary material for: Epigenetic modelling of former, current and never smokers
Source: Clin Epigenetics. 2021 Nov 17;13:206. doi: 10.1186/s13148-021-01191-6 (PMC8597260; doi:10.1186/s13148-021-01191-6)
Supplement: Supplementary file 2 — Additional file 2. Supplementary Tables 2A-2H - Supplementary Tables displaying results from enrichment analyses of genes mapped to CpGs from the smoking models presented in this paper. Supplementary Figures 1-4 - Network graphs of Human Phenotype Ontologies associated with CpGs from the smoking models presented in this paper. [file 13148_2021_1191_MOESM2_ESM.docx]

## A note on Additional file 2: Tables S2a-S2h and Additional file 2: Figures S1-S4

The Additional file 2: Tables below show results from enrichment analyses of genes mapped to CpGs in the smoking models presented in our paper. We looked at Human Phenotype Ontologies and BioPlanet 2019 (biological process) ontologies, respectively. Results are presented for all models, other than the candidate CpG LASSO current vs former smoking model, which simply did not show enrichment for any ontologies. Enrichr adjusts p-values by running a Fisher’s exact test for many random gene sets in order to compute a mean rank and SD from the expected rank for each term in the gene-set library, then calculates a z-score to assess deviation from the expected rank. Results in Additional file 2: Tables are presented as either all results with adjusted *P*<0.05, or, if no results have an adjusted *P*<0.05, all results with adjusted *P<0.1*. Finally, Additional file 2: Figures S1-S4 are network graphs designed to visualise how Human Phenotype Ontologies are associated with each other within the supplied gene-set. These graphs are not yet available for BioPlanet 2019 ontologies.

Additional file 2: Table S2a - Enrichment analysis results of Human Phenotype Ontology terms associated with genes mapped to CpGs in the ever/never agnostic smoking classifier

| Human Phenotype Ontology Term | Adjusted P-value | Odds Ratio |
| --- | --- | --- |
| Agitation (HP:0000713) | 2.73E-03 | 210.2 |
| Striae distensae (HP:0001065) | 2.73E-03 | 161.7 |
| Hypercortisolism (HP:0001578) | 2.73E-03 | 161.7 |
| Restlessness (HP:0000711) | 3.70E-03 | 116.7 |
| Truncal obesity (HP:0001956) | 4.66E-03 | 91.3 |
| Adrenal overactivity (HP:0002717) | 5.61E-03 | 75.0 |
| Lack of skin elasticity (HP:0100679) | 5.70E-03 | 65.6 |
| Depression (HP:0000716) | 5.70E-03 | 63.6 |
| Anxiety (HP:0000739) | 5.70E-03 | 60.0 |
| Psychosis (HP:0000709) | 7.59E-03 | 48.8 |
| Round face (HP:0000311) | 8.17E-03 | 44.6 |
| Thin skin (HP:0000963) | 9.77E-03 | 38.8 |
| Somatic mutation (HP:0001428) | 1.21E-02 | 33.3 |
| Osteopenia (HP:0000938) | 1.34E-02 | 30.4 |
| Osteoporosis (HP:0000939) | 1.44E-02 | 27.6 |
| Bruising susceptibility (HP:0000978) | 1.44E-02 | 27.2 |
| Recurrent fractures (HP:0002757) | 2.83E-02 | 18.5 |
| Enlarged penis (HP:0000040) | 3.30E-02 | 142.7 |
| Focal motor seizures (HP:0011153) | 3.30E-02 | 142.7 |
| Elevated circulating parathyroid hormone (PTH) level (HP:0003165) | 3.36E-02 | 124.8 |
| Menstrual irregularities (HP:0000858) | 3.36E-02 | 124.8 |
| Macroorchidism (HP:0000053) | 4.42E-02 | 83.2 |
| Growth hormone excess (HP:0000845) | 4.42E-02 | 83.2 |
| Hyperphosphatemia (HP:0002905) | 4.56E-02 | 76.8 |

Additional file 2: Figure S1 - Network graph of the top 10 Human Phenotype Ontologies enriched for genes mapped to ever/never CpGs in the agnostic smoking classifier


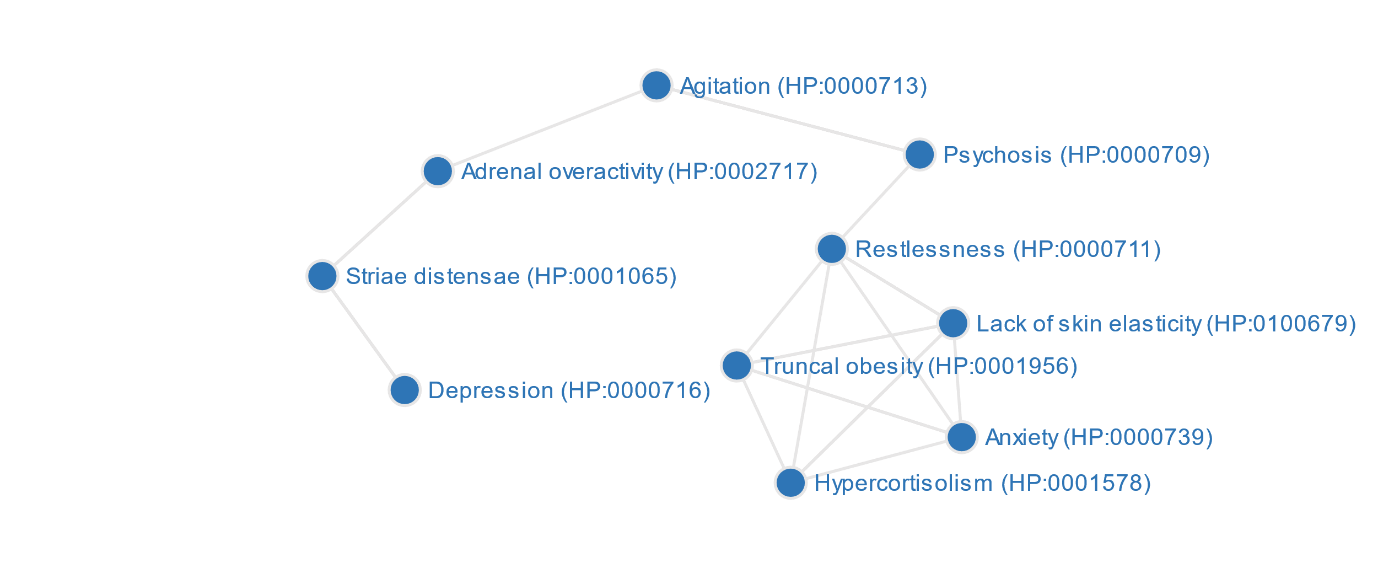


Additional file 2: Table S2b - Enrichment analysis results of BioPlanet 2019 terms (biological processes) associated with genes mapped to CpGs in the ever/never agnostic smoking classifier

| BioPlanet 2019 term | Adjusted P-value | Odds Ratio |
| --- | --- | --- |
| Hemostasis pathway | 9.47E-02 | 9.3 |
| Platelet homeostasis | 9.47E-02 | 24.9 |
| G alpha (s) signaling events | 9.47E-02 | 16.1 |
| Chromatin remodeling by nuclear receptors to facilitate initiation of transcription in carcinoma cells | 9.47E-02 | 135.9 |
| Rapid glucocorticoid receptor pathway | 9.47E-02 | 118.9 |
| Glycoprotein 1b-IX-V activation signaling | 9.47E-02 | 105.7 |
| Attenuation of GPCR signaling | 9.47E-02 | 95.1 |
| Valine, leucine and isoleucine biosynthesis | 9.47E-02 | 95.1 |
| Folate biosynthesis | 9.47E-02 | 95.1 |
| Beta-arrestins in GPCR desensitization | 9.47E-02 | 86.4 |
| Platelet adhesion to exposed collagen | 9.47E-02 | 79.2 |
| MAP kinase inactivation of SMRT corepressor | 9.47E-02 | 73.1 |
| CARM1 transcriptional regulation by protein methylation | 9.47E-02 | 73.1 |
| Coagulation intrinsic pathway | 9.47E-02 | 67.9 |
| LPA4-mediated signaling events | 9.47E-02 | 63.4 |
| Inhibition of platelet activation by aspirin | 9.47E-02 | 63.4 |
| Syndecan 3 pathway | 9.47E-02 | 59.4 |
| Beta-arrestin-dependent recruitment of Src kinases in GPCR signaling | 9.47E-02 | 59.4 |
| Ion channel function in vascular endothelium | 9.47E-02 | 59.4 |
| Corticosteroids and cardioprotection | 9.47E-02 | 55.9 |
| Transcriptional activity regulation by PML | 9.47E-02 | 55.9 |
| Serotonin receptor 4/6/7 and NR3C signaling | 9.47E-02 | 55.9 |
| PKA activation in glucagon signaling | 9.47E-02 | 55.9 |
| Platelet activation, signaling and aggregation | 9.47E-02 | 9.7 |
| GATA3-mediated activation of Th2 cytokine expression | 9.47E-02 | 50.0 |
| Estrogen receptor signaling pathway | 9.47E-02 | 50.0 |
| ChREBP regulation by carbohydrates and cAMP | 9.57E-02 | 47.5 |
| CCR3 signaling in eosinophils | 9.59E-02 | 45.3 |
| Progesterone-initiated oocyte maturation | 9.59E-02 | 43.2 |
| Cytosolic tRNA aminoacylation | 9.59E-02 | 41.3 |
| Wnt signaling pathway | 9.59E-02 | 8.6 |
| Ck1/Cdk5 regulation by type 1 glutamate receptors | 9.59E-02 | 39.6 |
| RXR/VDR pathway | 9.66E-02 | 38.0 |
| ADP signalling through P2Y purinoceptor 12 | 9.66E-02 | 36.5 |
| Nitric oxide stimulation of guanylate cyclase | 9.66E-02 | 35.2 |
| Retinoic acid receptor-mediated signaling | 9.66E-02 | 32.8 |
| FSH regulation of apoptosis | 9.66E-02 | 7.6 |
| Prothrombin activation intrinsic pathway | 9.66E-02 | 30.6 |
| Fibrin clot formation (clotting cascade) | 9.66E-02 | 30.6 |
| Glucagon signaling in metabolic regulation | 9.66E-02 | 29.7 |
| Glucagon-type ligand receptors | 9.66E-02 | 29.7 |
| N-cadherin signaling events | 9.66E-02 | 28.8 |
| Nuclear receptors in lipid metabolism and toxicity | 9.66E-02 | 28.8 |
| Ion transport by P-type ATPases | 9.90E-02 | 27.1 |
| Platelet aggregation (plug formation) | 9.90E-02 | 26.4 |
| Nuclear receptors | 9.90E-02 | 25.7 |
| E-cadherin nascent AJ-like junctions pathway | 9.90E-02 | 25.0 |
| Vitamin A and carotenoid metabolism | 9.90E-02 | 25.0 |

Additional file 2: Table S2c - Enrichment analysis results of Human Phenotype Ontology terms associated with genes mapped to CpGs in the current/former agnostic smoking classifier

| Human Phenotype Ontology Term | Adjusted P-value | Odds Ratio |
| --- | --- | --- |
| Axonal loss (HP:0003447) | 5.32E-02 | 219.5 |
| Opisthotonus (HP:0002179) | 5.32E-02 | 96.0 |
| Progressive neurologic deterioration (HP:0002344) | 5.32E-02 | 76.8 |
| Delayed gross motor development (HP:0002194) | 5.32E-02 | 76.8 |
| Abnormal social behavior (HP:0012433) | 5.32E-02 | 69.8 |
| Impaired social interactions (HP:0000735) | 5.32E-02 | 69.8 |
| Abnormality of purine metabolism (HP:0004352) | 5.32E-02 | 66.8 |
| Ectopia lentis (HP:0001083) | 5.32E-02 | 52.9 |
| Peripheral demyelination (HP:0011096) | 5.32E-02 | 52.9 |
| Abnormality of nucleobase metabolism (HP:0010932) | 5.57E-02 | 45.1 |
| Spastic tetraplegia (HP:0002510) | 5.92E-02 | 38.4 |
| Gliosis (HP:0002171) | 6.93E-02 | 27.9 |
| Full cheeks (HP:0000293) | 6.93E-02 | 27.4 |
| Nephrolithiasis (HP:0000787) | 7.04E-02 | 23.6 |
| Myoclonus (HP:0001336) | 7.04E-02 | 23.2 |
| Long face (HP:0000276) | 9.27E-02 | 16.3 |
| Hypoplasia of the corpus callosum (HP:0002079) | 9.44E-02 | 15.0 |

Additional file 2: Figure S2 - Network graph of the top 10 Human Phenotype Ontologies enriched for genes mapped to current/former CpGs in the agnostic smoking classifier


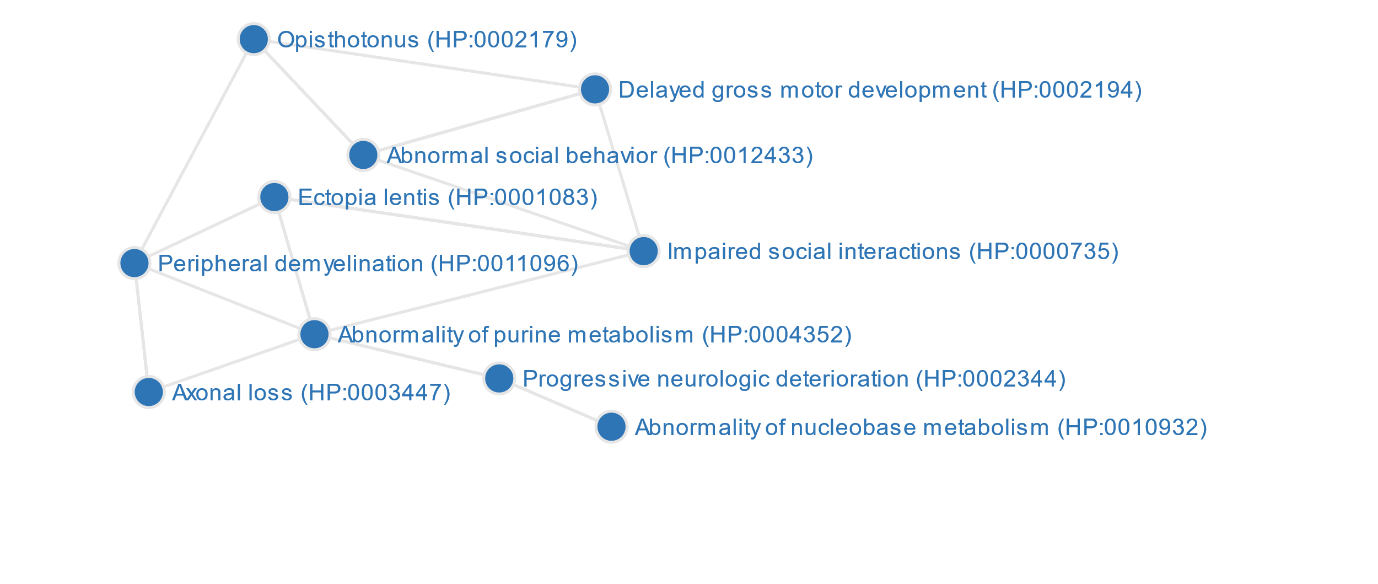


Table 3d - Enrichment analysis results of BioPlanet 2019 terms (biological processes) associated with genes mapped to CpGs in the current/former agnostic smoking classifier

| BioPlanet 2019 term | Adjusted P-value | Odds Ratio |
| --- | --- | --- |
| Molybdenum cofactor biosynthesis | 7.11E-02 | 307.4 |
| Facilitative sodium-independent glucose transporters | 7.11E-02 | 139.7 |
| Class C GPCRs (metabotropic glutamate and pheromone receptors) | 7.49E-02 | 85.3 |
|  |  |  |

Additional file 2: Table S2e - Enrichment analysis results of Human Phenotype Ontology terms associated with genes mapped to CpGs in the ever/never Guida smoking classifier

| Human Phenotype Ontology Term | Adjusted P-value | Odds Ratio |
| --- | --- | --- |
| B lymphocytopenia (HP:0010976) | 4.24E-02 | 237.9 |
| Abnormality of B cell number (HP:0010975) | 4.24E-02 | 208.1 |
| Neonatal short-limb short stature (HP:0008921) | 4.24E-02 | 208.1 |
| Natal tooth (HP:0000695) | 4.24E-02 | 166.5 |
| Mild short stature (HP:0003502) | 4.24E-02 | 151.3 |
| Abnormal number of incisors (HP:0011064) | 4.24E-02 | 151.3 |
| Conical tooth (HP:0000698) | 4.24E-02 | 118.9 |
| Cone-shaped epiphyses of the phalanges of the hand (HP:0010230) | 4.24E-02 | 104.0 |
| Facial cleft (HP:0002006) | 4.24E-02 | 104.0 |
| Abnormality of the alveolar ridges (HP:0006477) | 4.24E-02 | 97.9 |
| Complete atrioventricular canal defect (HP:0001674) | 4.24E-02 | 92.4 |
| Advanced eruption of teeth (HP:0006288) | 4.24E-02 | 92.4 |
| Cubitus valgus (HP:0002967) | 4.24E-02 | 79.2 |
| Synostosis of carpal bones (HP:0005048) | 4.24E-02 | 79.2 |
| Abnormality of the epiphyses of the phalanges of the hand (HP:0005920) | 4.24E-02 | 75.6 |
| Acute myeloid leukemia (HP:0004808) | 4.24E-02 | 75.6 |
| Abnormality of the incisor (HP:0000676) | 4.24E-02 | 72.3 |
| Atrioventricular canal defect (HP:0006695) | 4.24E-02 | 72.3 |
| Abnormality of the antihelix (HP:0009738) | 4.24E-02 | 72.3 |
| Abnormality of the epiphyses of the hand (HP:0005924) | 4.31E-02 | 66.5 |
| Abnormality involving the epiphyses of the upper limbs (HP:0003839) | 4.31E-02 | 64.0 |
| Emphysema (HP:0002097) | 4.37E-02 | 57.4 |
| Carpal synostosis (HP:0009702) | 4.37E-02 | 57.4 |
| Hypoplastic toenails (HP:0001800) | 4.54E-02 | 52.0 |
| Synostosis involving bones of the hand (HP:0004278) | 4.54E-02 | 48.9 |
| Postaxial foot polydactyly (HP:0001830) | 4.54E-02 | 46.2 |
| Hypotelorism (HP:0000601) | 4.54E-02 | 46.2 |
| Short ribs (HP:0000773) | 4.54E-02 | 44.9 |
| Short thorax (HP:0010306) | 4.95E-02 | 39.6 |

Additional file 2: Figure S3 - Network graph of the top 10 Human Phenotype Ontologies enriched for genes mapped to ever/never CpGs in the Guida smoking classifier


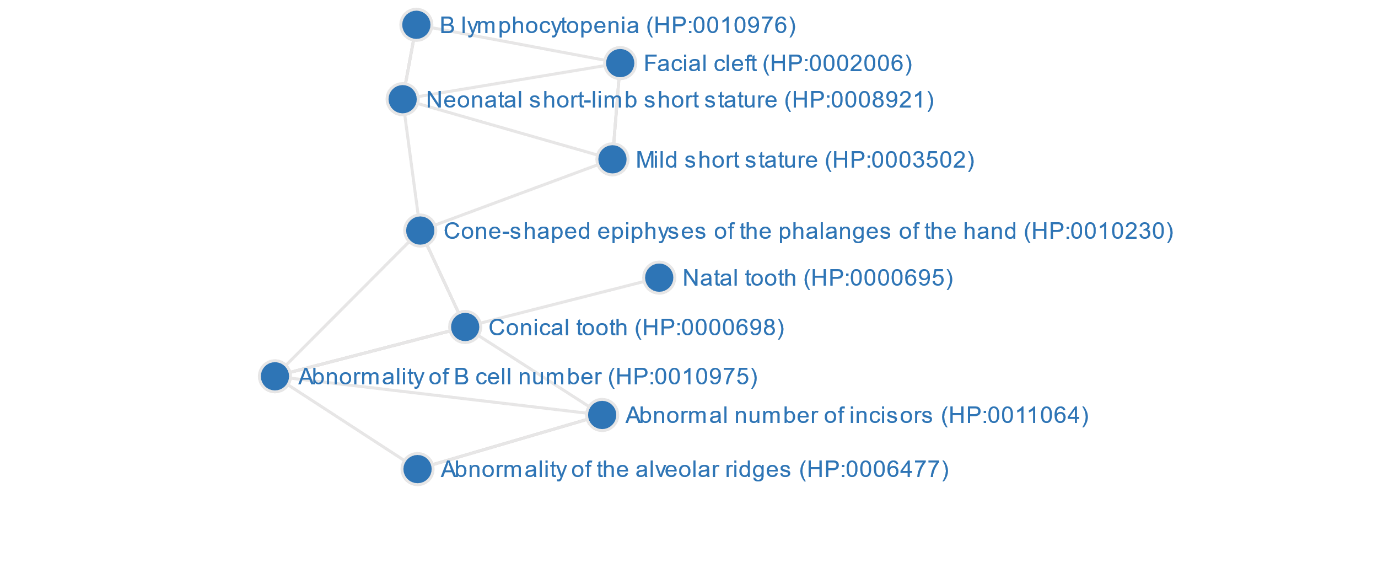


Table 3f - Enrichment analysis results of BioPlanet 2019 terms (biological processes) associated with genes mapped to CpGs in the ever/never Guida smoking classifier

| BioPlanet 2019 term | Adjusted P-value | Odds Ratio |
| --- | --- | --- |
| Thrombin signaling through protease-activated receptors | 3.08E-03 | 260.7 |
| Platelet activation, signaling and aggregation | 1.55E-02 | 65.0 |
| Gastrin-CREB signaling pathway via PKC and MAPK | 1.55E-02 | 64.7 |
| Folate biosynthesis | 2.18E-02 | 499.6 |
| Inhibition of platelet activation by aspirin | 2.18E-02 | 333.0 |
| GPCR ligand binding | 2.18E-02 | 32.0 |
| Hemostasis pathway | 2.18E-02 | 27.9 |
| Presynaptic function of kainate receptors | 2.18E-02 | 249.7 |
| ADP signalling through P2Y purinoceptor 12 | 2.18E-02 | 192.0 |
| G-protein activation | 2.18E-02 | 184.9 |
| G-protein beta-gamma signalling | 2.18E-02 | 184.9 |
| Inhibition of insulin secretion by adrenaline/noradrenaline | 2.18E-02 | 178.3 |
| Activation of kainate receptors upon glutamate binding | 2.18E-02 | 172.1 |
| Signal amplification | 2.18E-02 | 166.4 |
| Inwardly rectifying potassium channels | 2.18E-02 | 166.4 |
| Glucagon signaling in metabolic regulation | 2.18E-02 | 156.0 |
| Glucagon-type ligand receptors | 2.18E-02 | 156.0 |
| Insulin secretion regulation by glucagon-like peptide-1 | 2.65E-02 | 118.8 |
| G alpha (z) signaling events | 2.65E-02 | 113.4 |
| Aquaporin-mediated transport | 2.80E-02 | 101.8 |
| GABA A and B receptor activation | 2.82E-02 | 95.9 |
| G alpha (12/13) signaling events | 3.78E-02 | 65.5 |
| Opioid signaling | 3.78E-02 | 63.0 |
| Platelet homeostasis | 3.78E-02 | 61.5 |
| Signaling by GPCR | 3.78E-02 | 13.0 |
| Signal transduction | 3.78E-02 | 12.4 |
| G-protein signaling pathways | 3.78E-02 | 52.9 |
| Class B GPCRs (secretin family receptors) | 3.78E-02 | 52.9 |
| Potassium channels | 3.80E-02 | 50.8 |
| G alpha (s) signaling events | 4.48E-02 | 40.1 |
| Integration of energy metabolism | 4.48E-02 | 40.1 |

Additional file 2: Table S2g - Enrichment analysis results of Human Phenotype Ontology terms associated with genes mapped to CpGs in the Maas smoking classifier

| Human Phenotype Ontology Term | Adjusted P-value | Odds Ratio |
| --- | --- | --- |
| B lymphocytopenia (HP:0010976) | 5.62E-03 | 713.9 |
| Abnormality of B cell number (HP:0010975) | 5.62E-03 | 624.6 |
| Acute myeloid leukemia (HP:0004808) | 9.56E-03 | 227.0 |
| Neutropenia (HP:0001875) | 1.80E-02 | 87.4 |
| Autosomal dominant inheritance (HP:0000006) | 2.53E-01 | 4.16 |

Additional file 2: Figure S4 - Network graph of Human Phenotype Ontologies enriched for genes mapped to CpGs in the Maas smoking classifier


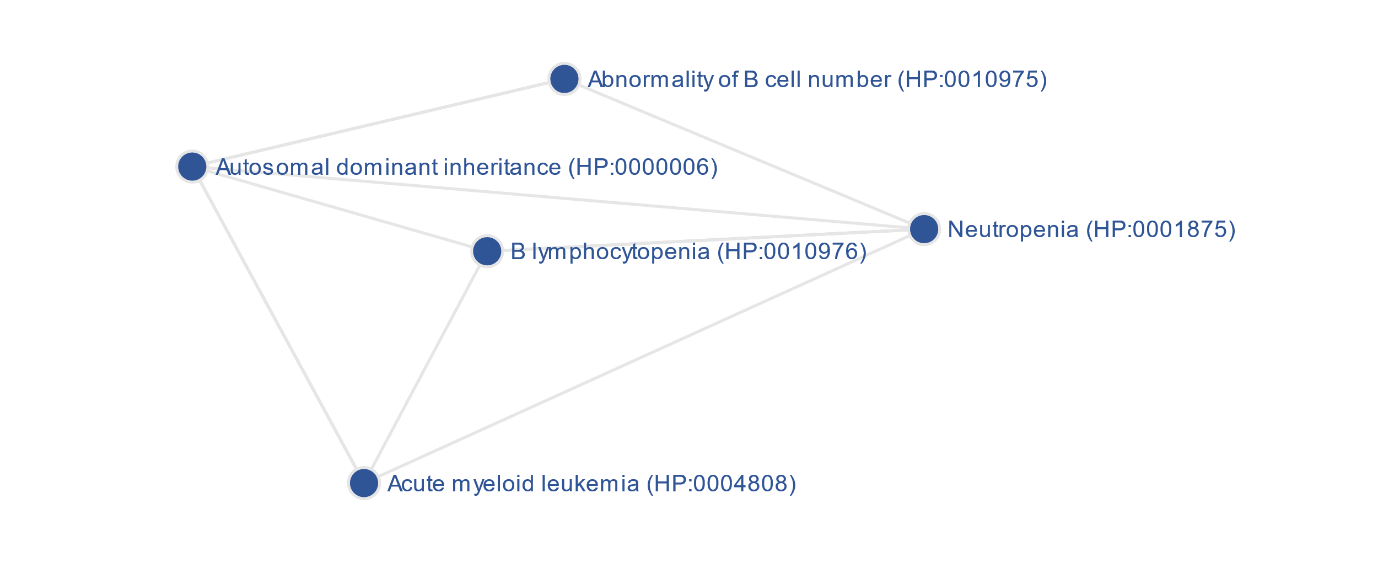


Table 3h - Enrichment analysis results of BioPlanet 2019 terms (biological processes) associated with genes mapped to CpGs in the Maas smoking classifier

| BioPlanet 2019 term | Adjusted P-value | Odds Ratio |
| --- | --- | --- |
| Inhibition of platelet activation by aspirin | 5.19E-02 | 333.0 |
| Thrombin signaling through protease-activated receptors | 7.85E-02 | 95.9 |
| Myc repressed pathway | 7.85E-02 | 69.2 |
| TNF-alpha effects on cytokine activity, cell motility, and apoptosis | 9.37E-02 | 37.1 |
| Peptide G-protein coupled receptors | 9.37E-02 | 25.9 |
| Platelet activation, signaling and aggregation | 9.37E-02 | 24.3 |
| Gastrin-CREB signaling pathway via PKC and MAPK | 9.37E-02 | 24.1 |
| Class A GPCRs (rhodopsin-like) | 9.56E-02 | 19.6 |
| Neuroactive ligand-receptor interaction | 9.56E-02 | 18.2 |
