# Supplementary material for: Epigenetic modelling of former, current and never smokers
Source: Clin Epigenetics. 2021 Nov 17;13:206. doi: 10.1186/s13148-021-01191-6 (PMC8597260; doi:10.1186/s13148-021-01191-6)
Supplement: Supplementary file 3 — Additional file 3. Supplementary Figure 5 - Comparison of the discriminative ability of cotinine and DNA methylation when distinguishing former from never smokers. [file 13148_2021_1191_MOESM3_ESM.docx]

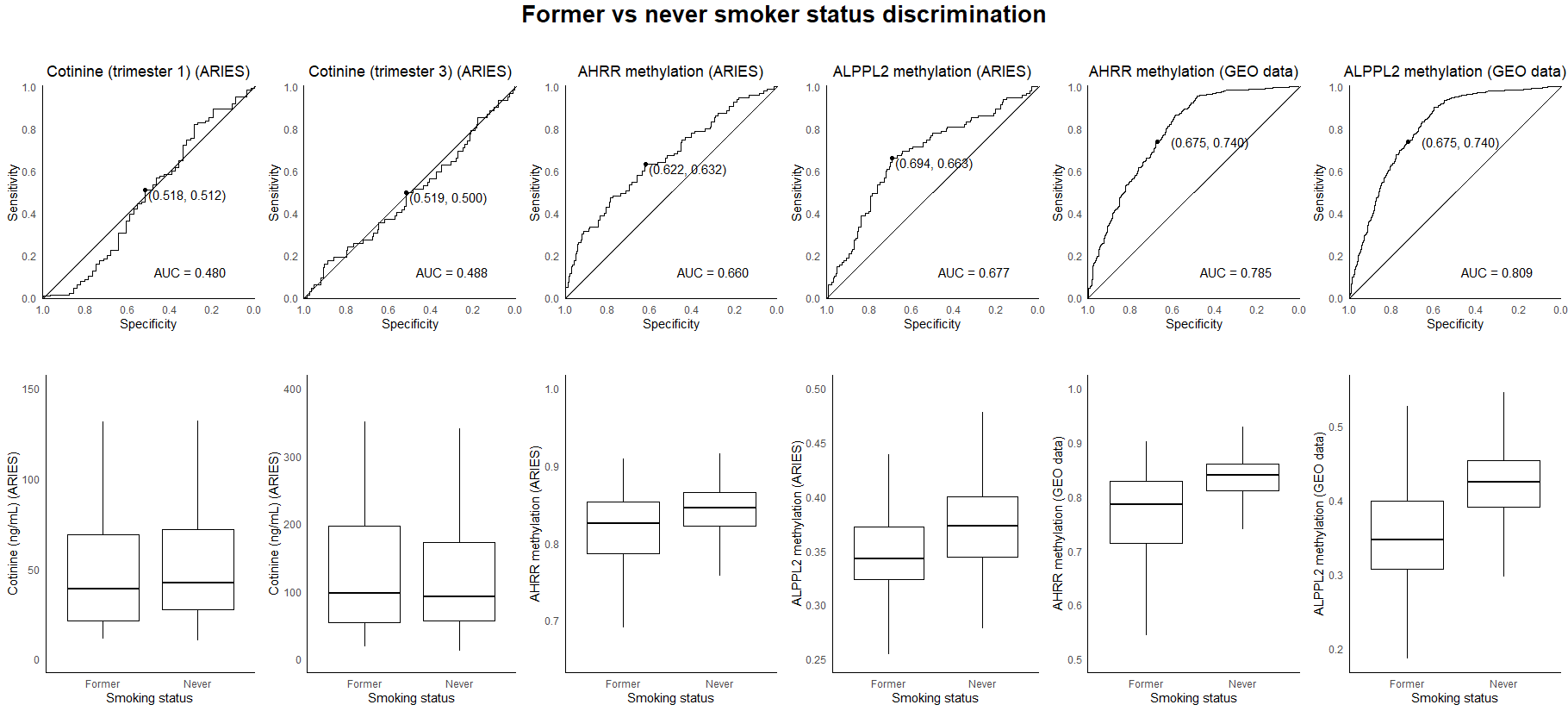


Additional file 3: Figure S5 - Comparison of the discriminative ability of cotinine and DNA methylation when distinguishing former from never smokers. ROC curves and boxplots are shown for each biomarker. Data are ordered by AUC, from left to right. Cotinine and DNA methylation measurements were available in ARIES (columns 1-5). AUCs can also be seen for the data used in this study (columns 6-7), where only DNAm was available.
